# Exploiting 2D Neural Network Frameworks for 3D Segmentation Through Depth Map Analytics of Harvested Wild Blueberries (*Vaccinium angustifolium* Ait.)

**DOI:** 10.3390/jimaging10120324

**Published:** 2024-12-15

**Authors:** Connor C. Mullins, Travis J. Esau, Qamar U. Zaman, Ahmad A. Al-Mallahi, Aitazaz A. Farooque

**Affiliations:** 1 Department of Engineering, Faculty of Agriculture, Dalhousie University, Truro, NS B2N 5E3, Canada; 2 Faculty of Sustainable Design Engineering, University of Prince Edward Island, Charlottetown, PE C1A 4P3, Canada; 3 Canadian Center for Climate Change and Adaptation, University of Prince Edward Island, St Peters Bay, PE C0A 2A0, Canada

**Keywords:** Detectron2, YOLOv8, point clouds, time of flight, precision agriculture

## Abstract

This study introduced a novel approach to 3D image segmentation utilizing a neural network framework applied to 2D depth map imagery, with Z axis values visualized through color gradation. This research involved comprehensive data collection from mechanically harvested wild blueberries to populate 3D and red–green–blue (RGB) images of filled totes through time-of-flight and RGB cameras, respectively. Advanced neural network models from the YOLOv8 and Detectron2 frameworks were assessed for their segmentation capabilities. Notably, the YOLOv8 models, particularly YOLOv8n-seg, demonstrated superior processing efficiency, with an average time of 18.10 ms, significantly faster than the Detectron2 models, which exceeded 57 ms, while maintaining high performance with a mean intersection over union (IoU) of 0.944 and a Matthew’s correlation coefficient (MCC) of 0.957. A qualitative comparison of segmentation masks indicated that the YOLO models produced smoother and more accurate object boundaries, whereas Detectron2 showed jagged edges and under-segmentation. Statistical analyses, including ANOVA and Tukey’s HSD test (α = 0.05), confirmed the superior segmentation performance of models on depth maps over RGB images (*p* < 0.001). This study concludes by recommending the YOLOv8n-seg model for real-time 3D segmentation in precision agriculture, providing insights that can enhance volume estimation, yield prediction, and resource management practices.

## 1. Introduction

**Figure 1:**
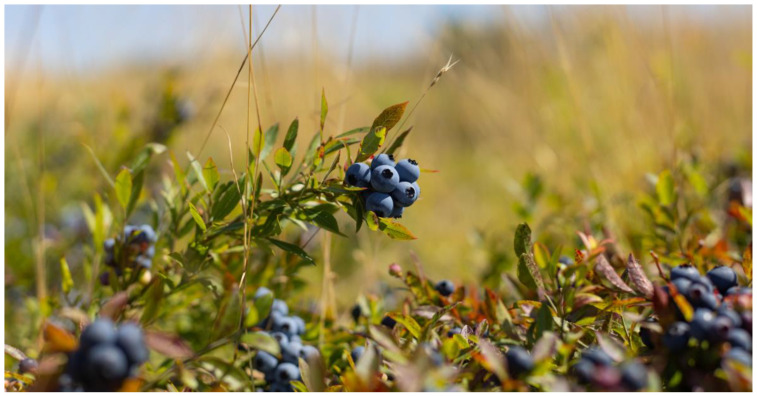
Example of wild blueberries (*Vaccinium angustifolium* Ait.) at time of harvest, illustrating the irregular clustering.

The fusion of computational methods with traditional farming practices in the cultivation of wild blueberries (*Vaccinium angustifolium* Ait.) has led to increased efficiency and precision in cultivation [1,2]. Wild blueberries (Figure 1) are a crop that presents distinct challenges due to their unique field conditions and harvesting requirements. Unlike cultivated (*Vaccinium corymbosum* L.) varieties, wild blueberries grow without planting or seeding, often in rugged, uneven terrains [3,4]. This non-uniform setting contributes to the complexity of their mechanical harvesting, making harvesting picker head implement height adjustments frequent.

The use of advanced computer vision techniques, such as point cloud segmentation, explored in this research can address the unique challenges faced by wild blueberry harvester operators in ensuring consistent and full collection totes. Historically, 3D vision research in agriculture has predominantly overlooked specialty crops like wild blueberries [5,6,7,8]. Harvesting wild blueberries is complex due to their varied sizes and shapes and natural clustering. These factors, combined with irregular field conditions, pose significant challenges for image processing and volume estimation. Conventional 3D vision approaches, such as RGB meshed segmentation or clustering-based segmentation [7,9,10], lack the depth and volume information crucial for accurately processing irregular and densely clustered objects like wild blueberries. In contrast, the segmentation of depth maps for 3D point clouds, as proposed in this research, offers a more comprehensive analysis by incorporating depth information, which is vital for precise volume estimation and spatial analysis.

To optimize the point cloud volume estimation of harvested wild blueberries, segmenting outlier points is crucial for improving the accuracy of volume estimation algorithms [5,11]. Accurate volume estimation is crucial for several reasons: it directly influences yield assessment, aids in quality control through the categorization of harvested berries based on volume (indicating maturity and quality) [12], and offers logistical benefits such as optimizing storage and transportation [13]. Subsequently, accurate volume estimation supports yield prediction and the generation of prescription maps, enhancing crop management through precision irrigation, fertilization, and drainage [14]. A critical step for accurate volume estimation is the effective segmentation of the tote containing the berries from the point cloud data. This involves distinguishing the berries from their storage tote and any other surrounding objects or noise within the 3D scan. This step is challenging due to the irregular and varying shape of the berries [15], variability in fill shapes, and other elements within the tote. Effective segmentation ensures that the volume computation applies only to the berries, excluding the tote and any extraneous elements. To use point cloud analytics for volume estimation, one study on the volume estimation of tree canopies using LiDAR highlighted three methods for volume estimation: convex hull, alpha shape, and voxel grid, finding the superior performance of convex hull and alpha shape over voxel grid [16].

Point cloud segmentation is crucial in enhancing the precision of volume estimation. Inaccurate segmentation could lead to the overestimation or underestimation of berry volume, causing inefficiencies in processing and potential economic losses [17,18]. Moreover, precise volume estimation of harvested wild blueberries using 3D point cloud data has broader implications, paving the way for automated systems that streamline the harvesting process, reduce manual labor, and enhance accuracy and efficiency. This is particularly beneficial for crops like wild blueberries, where the harvesting and processing are labor-intensive and subject to human error.

Neural network frameworks like You Only Look Once (YOLO) have effectively segmented 2D images with high precision [19,20], enabling the removal of unwanted points from generated depth maps. Ultralytics’ YOLOv8 (Ultralytics LLC, Los Angeles, CA, USA) marks a significant improvement in the YOLO series, surpassing previous versions in speed and accuracy (v7n~15ms, v8n~5ms) [20]. YOLOv8’s core architecture, based on YOLOv7 ELAN design principles [21], features the innovative C2f module, which enhances gradient flow for robust performance [21]. It also incorporates spatial pyramid pooling fusion, crucial for extracting contextual data from various image scales, boosting the model’s ability to generalize. Additionally, YOLOv8’s neck design eliminates convolutional layers in the up-sampling process and replaces the C3 module with the C2f module for optimized efficiency [21]. YOLOv8 offers a comprehensive training framework supporting key functionalities like object detection and instance segmentation [22,23].

Detectron2 is an open-source library tailored for computer vision tasks, including object detection, segmentation, and pixel-wise predictions [24,25]. Developed using Python and built on the PyTorch framework, it allows for the dynamic creation and debugging of neural networks. The library features a comprehensive model zoo with pre-trained models on datasets such as COCO, LVIS, and Cityscapes. Detectron2 supports multi-GPU training and mixed precision to accelerate processes. The architecture comprises several key components: the backbone, neck, and heads. The backbone extracts features from images, supporting architectures like ResNet, ResNeXt, and EfficientNet [26]. The neck, featuring components like the Feature Pyramid Network (FPN), processes these features for multi-scale object detection. The heads are tailored to specific tasks (R-CNN for object detection, Mask R-CNN for instance segmentation), predicting outputs such as bounding boxes and segmentation masks. This structured approach makes Detectron2 a powerful and flexible framework for complex visual recognition tasks. In instance segmentation, the ResNet 50 and 101 (R50 and R101) models using FPN achieved the best mAP scores within their model depths on the COCO dataset [26]. However, the ResNeXt 101 (X101) achieved the highest overall mAP score but had nearly twice the processing time of the ResNet-101 FPN model and more than twice the processing time of the ResNet-50 FPN model [26].

The objective of this research was to conduct a comparative analysis of the YOLOv8-seg and Detectron2 models to determine the most effective method for point cloud segmentation in the context of harvested wild blueberry totes. This comparison was crucial for understanding how different deep learning architectures perform in a specialized agricultural setting, especially one that involves complex and irregular natural objects. The models were assessed based on their accuracy, efficiency, and ability to handle nuances in the dataset, such as the differentiation between berries and the tote. The innovation of this study lies in applying 2D neural networks to segment 3D point clouds through depth map conversion in real time. The comparison of RGB-based segmentation and depth map segmentation will showcase the superior performance of this innovative method.

## 2. Materials and Methods

**Figure 2:**
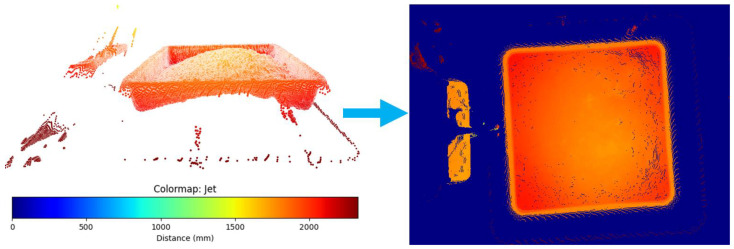
Visual demonstration of conversion from point cloud to depth map using the jet colormap as *Z* axis representation in mm, where the background color of the depth map was set to blue.

**Figure 3:**
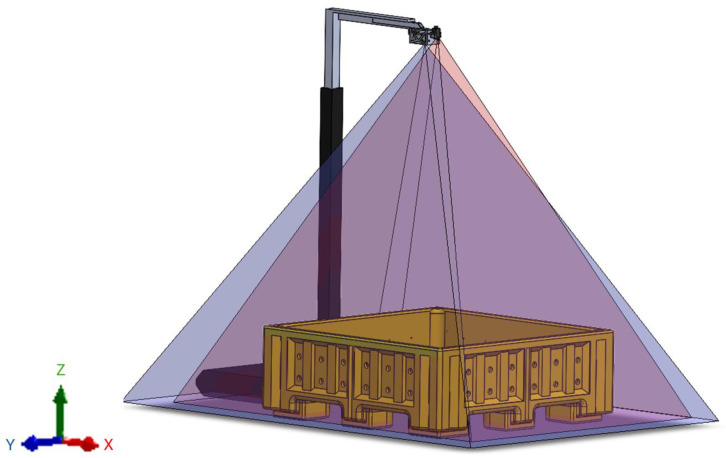
Dual camera mount setup for data collection with Basler Blaze-101 (67° by 51° in the X and Y axes, respectively) and Lucid Vision Labs Triton (60° by 46° in the X and Y axes, respectively).

Neural network frameworks were deployed on 2D depth map imagery, where the Z axis values were represented by color (Figure 2) (axes shown in Figure 3). The experiment for evaluating the efficacy of this newly proposed technique required data of varying shapes within a specified storage tote filled with mechanically harvested wild blueberries.

### 2.1. Data Collection

Mechanically harvested wild blueberry totes (1.12 m × 1.12 m × 0.21 m) were offloaded and then transported with a John Deere 5420 tractor with a 542 loader (John Deere, Moline, IL, USA) and pallet forks to the platform with above-mounted cameras (Figure 3). The point clouds and RGB images were collected after the tote was positioned so that each edge of the tote was aligned with the platform. A Blaze-101 ToF camera (Basler AG, Ahrensburg SH, Germany) with a field of view of 67° × 51° (in the X and Y axes, respectively) was mounted alongside a 1.6 MP Triton RGB camera (Lucid Vision Labs, Richmond BC, Canada) with a 4 mm lens (Edmund Optics, Barrington NJ, USA) directly over the platform using an extendable mount to allow point cloud stitching (Figure 3).

An MSI Workstation laptop (WS65 9TM1410CA, Micro-Star International Co., Ltd., New Taipei, Taiwan) with an Intel Core i9-9980H central processing unit (CPU, Intel Corporation, Santa Clara, CA, USA) and an Nvidia RTX Quadro 5000 graphics processing unit (GPU, Nvidia Corporation, Santa Clara, CA, USA) was used to collect the data from the ToF camera through a 10 m M12 to RJ45 cable (Basler AG, Ahrensburg SH, Germany) and connected to power using a 10 m M12 to GPIO cable (Basler AG, Ahrensburg SH, Germany). To collect the data, during the harvest of wild blueberries, the filled totes were carefully positioned beneath the RGB and ToF cameras elevated 1.35 m above the tote. The cameras captured the scenes and saved them as JPEG images with a resolution of 1440 × 1080 and Polygon files (PLY) with a resolution of 640 × 480. After image acquisition, JPEGs were uploaded to the Roboflow API [27]. The labeling of both RGB images and the depth map images was initially conducted using the Segment Anything Model [28], after which, manual adjustments were made as needed by selecting pre-allocated anchor points that over- or under-segmented the image and moving their position in the X or Y axis to outline the segmentation mask properly.

### 2.2. Framework Training and Evaluation

The dataset (380 images) was partitioned into distinct subsets to facilitate effective training and unbiased evaluation. Specifically, 80% of the data was allocated for training and the remaining data were split evenly between testing and validation, each receiving 10% [29]. This distribution ensured that the model was tested on unseen data and validated to prevent overfitting and confirm generalization. The models were trained on an Alienware Aurora R11 desktop computer (Dell Inc., Round Rock, TX, USA) with a 3.7 GHz 10-core Intel Core i9-10900K CPU (Intel Corporation, Santa Clara, CA, USA), 128 GB of DDR4-3200 MHz, and an Nvidia GeForce RTX 3090 GPU (Nvidia Corp., Santa Clara, CA, USA) at 150 epochs, with an early stopping patience of 50 and a batch size of 16.

During validation, a suite of methods and metrics were applied to quantify model performance comprehensively. Intersection over Union (IoU) was used due to its relevance in object detection and segmentation tasks [30]. Additionally, the Matthew’s correlation coefficient (MCC) was employed as it is particularly useful compared to F1 scores when dealing with imbalanced datasets without compromising results on balanced datasets [31]. MCCs provided a robust indication of pixel-wise classification accuracy by incorporating true and false positives and negatives, thus delivering a balanced metric that reflected both the presence and absence of the target condition effectively.

To prepare the data for evaluation in the 3D space, manually segmenting the point clouds to only contain the targeted tote contents while retaining the outermost points on each axis was required to supply the ground truth point clouds without altering the depth map. The ground truth point clouds were then converted to depth map format for model training and evaluation. This was done using both Python (3.8.13) and Meshlab (Visual Computing Lab, ISTI-CNR, Pisa, Italy), where Python was used to generate the depth maps for the data, training, and testing of the models and Meshlab was used for the manual segmentation of the point clouds.

Hue adjustment was the chosen augmentation technique during training to address the variability in color representations and ensure robustness to different color maps (cmaps) [32]. By modifying the hue values of the input images, the model could learn to be invariant to color shifts and changes, simulating a range of height conditions and camera settings. This form of color augmentation is critical in scenarios where the model is expected to operate in environments with varying distances from the cameras, where color is proportional to distance, or when the data are sourced from multiple devices with different profiles, such as the depth maps compared to RGB images [33,34].

### 2.3. Statistical Methods

To assign a random fill weight to the totes for generalization and statistical strength, the operator was instructed to drop the tote at the end of each side of the pass or until it was filled. As the operator moved through the field, they filled the tote with berries using the mechanical harvester. At the end of each pass across the field or when the tote was full, it was dropped, ensuring that the totes had varying fill weights, which was crucial to accurately represent the variability in berry volume and strengthen the statistical analysis of the data. A completely randomized design (CRD) was then incorporated; the deep learning framework model was the factor of interest, with the response variable being the segmentation mask, which was then evaluated using Analysis of Variance (ANOVA) and Tukey’s Honest Significant Difference (HSD) test for multiple mean comparisons on IoU and MCCs through Minitab 21 (Minitab, LLC, Minitab 21, State College, PA, USA.) at a significance threshold of α = 0.05.

## 3. Results and Discussion

The RGB and depth image masks were overlayed on the mask of the respective ground truth segmented images. The true positive (TP) and negative (TN) predictions were compared to false positive (FP) and false negative (FN) predictions to determine the efficacy of its use for segmentation (Table 1). It was clear that in both MCCs and IoU, the RGB meshed point cloud segmentation did not yield promising results for segmentation in 3D, evidenced by each model performing under 82% on both IoU and MCCs when compared to the models segmenting depth maps, performing consistently above 92%. Furthermore, the statistical analysis through multiple mean comparisons showed the separation between RGB and the depth-based networks, showing a statistically significant difference between them (*p* < 0.001).

**Figure 4:**
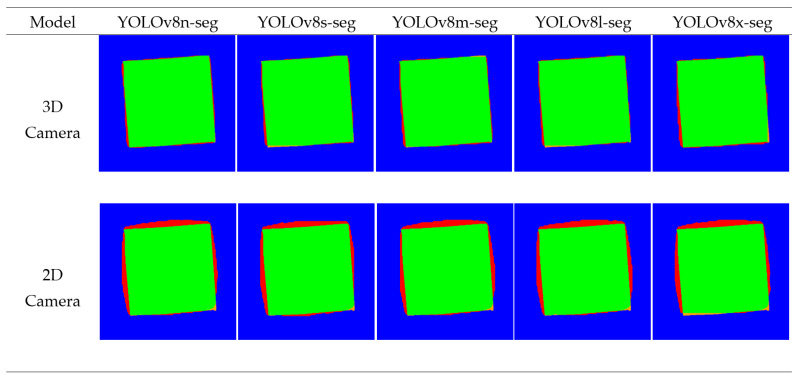
Visualization of segmentation mask correctness of YOLO masks for ToF 3D camera and 2D RGB camera, with true positive as green, true negative as blue, false positive as red, and false negative as orange.

**Figure 5:**
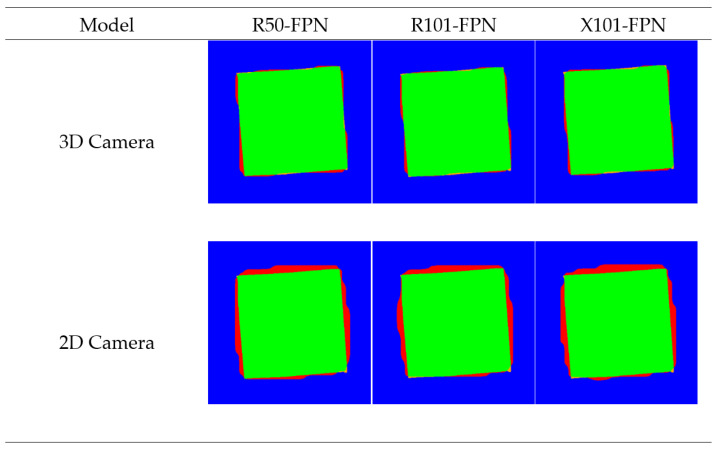
Visualization of segmentation mask correctness of Detectron2 masks for ToF 3D and 2D RGB cameras, with true positive as green, true negative as blue, false positive as red, and false negative as orange.

In assessing the models based on the framework, the Detectron2 models each displayed no significant differences in IoU and MCCs within the respective spatial domains (2D and 3D) (Table 1). There were, however, significant differences between each of the models based on processing time, where the lightweight ResNet50 was significantly faster than both the ResNet101 and ResNeXt101 models, with the ResNet101 model only being significantly faster than ResNeXt101 in the depth map prediction medium. Like the Detectron2 models, within each spatial domain, the YOLO variants showed no difference in MCCs and IoU. Both the YOLO and Detectron2 models achieved significantly superior performance using the depth maps as the prediction medium across each of the performance metrics. As a qualitative evaluation, the losses were visualized by overlaying the ground truth and predicted masks color-coded based on pixel correctness (TN, TP, FN, FP) (Figure 4 and Figure 5).

In the qualitative analysis of model performance, the segmentation masks generated by the various YOLO and Detectron2 models were compared to the ground truth masks (Figure 4 and Figure 5), focusing on the true positive (TP), true negative (TN), false positive (FP), and false negative (FN) values, each distinguished by colors: green, blue, red, and orange, respectively (Figure 4 and Figure 5). The visual aspect of color-coded segmentation outputs, as presented in Figure 4 and Figure 5, provided a powerful tool for evaluating model performance. By assigning distinct colors to the categorized pixels, the visual overlays offered an intuitive and immediate understanding of segmentation accuracy. This approach allowed for error pattern identification, such as areas with high misclassification rates, and assessed the smoothness and precision of segmentation boundaries.

This comparison revealed distinct patterns and errors in each model’s performance. The errors associated with the RGB level segmentations could be primarily attributed to the camera lens warp causing misalignment as the lens used in this study had a recorded maximum distortion of −20.43%. Both the YOLOv8-seg and Detectron2 models tended to under-segment the background imagery, leading to more FPs proportionally. The Detectron2 models’ under-segmentation in comparison to the YOLO models was less smooth, where the images segmented by the Detectron2 model had jagged and irregular boundaries between segments, indicating that the model struggled to create clean and precise edges (Figure 5). This lack of smoothness led to inaccuracies in segmenting the target objects, as the rough edges could either include or exclude adjacent areas incorrectly. Opposing this, the segmentation output from the YOLO models showed smoother boundaries, where the edges were more consistent and less jagged, which indicated that the YOLO models could delineate objects with higher precision (Figure 4). Smoother segmentation resulted in a more accurate representation of the objects’ shapes and sizes, which is critical for tasks requiring precise volume estimation, such as in agricultural applications for yield prediction.

**Figure 6:**
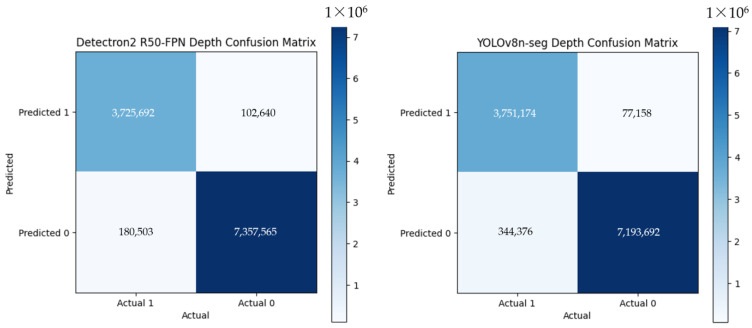
Sample confusion matrices of Detectron2 R50 with FPN and YOLOv8n-seg on the testing dataset of the depth image dataset.

**Figure 7:**
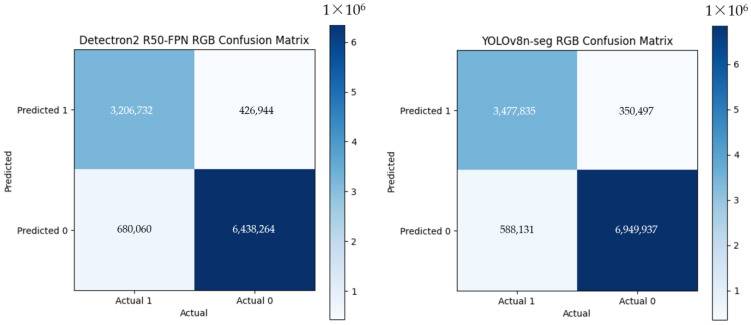
Sample confusion matrices of Detectron2 R50 with FPN and YOLOv8n-seg on the testing dataset of the RGB image dataset.

The confusion matrices for depth-based and RGB-based segmentation revealed the performance of the YOLOv8 and Detectron2 frameworks, highlighting the strengths and weaknesses of each approach (Figure 6 and Figure 7). Depth-based segmentation outperformed RGB-based methods, achieving higher true positive (TP) and true negative (TN) rates (Figure 6). For depth-based segmentation, Detectron2 achieved TP = 3,725,692 and TN = 7,357,565, while YOLOv8n-seg recorded TP = 3,751,174 and TN = 7,193,692. YOLOv8n-seg also showed fewer false positives (FP = 77,158) and false negatives (FN = 344,376) compared to Detectron2 (FP = 102,640, FN = 180,503). This accuracy, combined with YOLOv8n-seg’s faster processing time (18.10 ms vs. Detectron2’s 57.67 ms), highlights its suitability for real-time agricultural applications.

In contrast, RGB-based segmentation performed worse, with increased error rates (Figure 7). Detectron2 achieved TP = 3,206,732 and TN = 6,438,264, while YOLOv8n-seg achieved higher values of TP = 3,477,835 and TN = 6,949,937. However, both frameworks recorded significantly higher false positives and false negatives, with Detectron2 at FP = 426,944 and FN = 680,060 and YOLOv8n-seg at FP = 350,497 and FN = 588,131. The lack of depth information and lens distortions (up to −20.43%) further hindered RGB-based segmentation. While YOLOv8n-seg processed images faster (28.42 ms vs. Detectron2′s 95.68 ms), RGB segmentation remained inadequate for precision tasks in the 3D space.

Based on both the quantitative and qualitative analyses, YOLOv8n-seg is recommended as a general segmentation solution for real-time applications using ToF imagery. However, for performance in the RGB domain specifically, the Detectron2 ResNet50 model is recommended for its IoU (0.785) and MCC (0.815). In real-time applications, the YOLOv8n-seg model is recommended for both mediums, with a significantly lower processing time (depth: 18.10 ms, RGB: 28.42 ms) and no significant difference in accuracy metrics (IoU: 0.944 and MCC: 0.957). YOLOv8 outperforms Detectron2 due to fundamental architectural differences. YOLOv8 employs a single-stage design, directly predicting segmentation outputs from feature maps without relying on a region proposal network (RPN). This streamlined approach reduces computational overhead and processing time while maintaining high accuracy. In contrast, Detectron2’s two-stage architecture, which uses an RPN and separate steps for detection and segmentation, introduces additional latency and increases computational complexity. YOLOv8 further benefits from adaptive anchor-free mechanisms and dynamic loss weighting, enabling the better handling of irregularly shaped objects and varying scales, such as those found in the context of wild blueberries. Its optimized convolutional layers and attention mechanisms produce smoother segmentation boundaries and reduce errors, as observed in the qualitative analysis. Detectron2, while robust, relies heavily on its FPN for multi-scale feature aggregation, which adds to its processing time and limits real-time applicability.

The results highlighted the unique strength of leveraging 2D neural network frameworks for depth map segmentation as a novel methodology for addressing 3D segmentation challenges. By utilizing 2D frameworks to analyze depth map imagery, which inherently encodes spatial information, this study demonstrated significant advancements in segmentation accuracy and processing speed, reinforcing the method’s potential as a practical solution for real-time precision agriculture applications.

## 4. Conclusions

The objective of this study was to evaluate the performance of the YOLOv8 and Detectron2 frameworks in segmenting RGB and depth images, comparing their segmentation efficacy and processing times in 3D image contexts to find an optimal solution for instance segmentation in the 3D space.

The analysis revealed that depth map-based segmentation models significantly outperformed RGB-based models in terms of both Intersection over Union (IoU) and Matthew’s correlation coefficient (MCC). Depth-based models consistently achieved IoU and MCC scores above 92%, while RGB-based models scored below 82%. For example, the YOLOv8s model achieved the highest IoU (0.952 ± 0.014) and MCC (0.963 ± 0.011) among the depth-based models, demonstrating superior segmentation accuracy. In contrast, the best-performing RGB model, YOLOv8l, managed an IoU of 0.810 ± 0.073 and an MCC of 0.779 ± 0.068. Comparing the two frameworks, both YOLOv8 and Detectron2 showed no significant differences in IoU and MCCs within their respective spatial domains (2D and 3D). However, the YOLOv8 models were significantly faster in their processing times. YOLOv8n achieved a processing time of 18.10 ± 3.68 ms, whereas the lightweight Detectron2 model, R50-FPN, was significantly slower (57.67 ± 7.35 ms). This difference in processing speed highlights YOLOv8’s advantage in real-time applications. In the qualitative analysis of model performance, segmentation masks generated by the YOLOv8 and Detectron2 models were compared to ground truth masks through true positive (TP), true negative (TN), false positive (FP), and false negative (FN) values. Both the YOLOv8-seg and Detectron2 models exhibited under-segmentation, where Detectron2’s output had jagged boundaries, indicating struggles with edge precision, whereas the YOLO models showed smoother, more accurate delineation of objects.

Future research should explore the application of segmentation models for improved volume estimation of 3D images. Integrating segmentation with advanced volume estimation techniques could enhance precision agriculture, enabling more accurate and efficient monitoring of harvesting volume and transportation optimization. Further studies should also investigate the performance of these models in different agricultural contexts and under varied environmental conditions, providing a more comprehensive evaluation of their capabilities and limitations.

## Figures and Tables

**Table 1:** YOLOv8 and Detectron2 model evaluation results for both RGB and depth map models (α = 0.05).

	Model	IoU	MCC	Processing Time (ms)
Depth	YOLOv8n	0.944 (±0.014) a	0.957 (±0.011) a	18.10 (±3.68) h
	YOLOv8s	0.952 (±0.014) a	0.963 (±0.011) a	20.52 (±6.98) h
	YOLOv8m	0.946 (±0.013) a	0.959 (±0.011) a	21.65 (±3.76) h
	YOLOv8l	0.943 (±0.015) a	0.956 (±0.012) a	22.20 (±3.68) g h
	YOLOv8x	0.921 (±0.033) a	0.938 (±0.027) a	29.29 (±3.77) f
	R50-FPN	0.945 (±0.013) a	0.958 (±0.010) a	57.67 (±7.35) e
	R101-FPN	0.951 (±0.014) a	0.963 (±0.011) a	64.44 (±5.86) d
	X101-FPN	0.948 (±0.013) a	0.960 (±0.010) a	81.82 (±5.12) c
RGB	YOLOv8n	0.780 (±0.068) b	0.811 (±0.073) b	28.42 (±3.87) f g
	YOLOv8s	0.780 (±0.066) b	0.810 (±0.071) b	30.91 (±9.42) f
	YOLOv8m	0.770 (±0.141) b	0.808 (±0.130) b	32.77 (±9.40) f
	YOLOv8l	0.810 (±0.073) b	0.779 (±0.068) b	33.51 (±8.75) f
	YOLOv8x	0.777 (±0.071) b	0.808 (±0.076) b	34.71 (±6.29) f
	R50-FPN	0.785 (±0.065) b	0.815 (±0.070) b	95.68 (±16.42) b
	R101-FPN	0.785 (±0.066) b	0.815 (±0.071) b	102.41 (±10.45) a
	X101-FPN	0.765 (±0.071) b	0.796 (±0.077) b	102.88 (±13.15) a

Means that do not share a letter are significantly different. Comparisons are within the column only.

## Data Availability

Data will be made available upon request.
